# Reflections on mental health research in post-disaster settings

**DOI:** 10.1371/journal.pgph.0005312

**Published:** 2025-10-14

**Authors:** Ahlke Kip, Gülşah Kurt, Ceren Acartürk, Philip Hyland, Dana Churbaji, Lasse B. Sander

**Affiliations:** 1 Medical Psychology and Medical Sociology, Faculty of Medicine, University of Freiburg, Freiburg, Germany; 2 School of Psychology, UNSW, Sydney, Australia; 3 Department of Psychology, Koc University, Istanbul, Türkiye; 4 Department of Psychology, Maynooth University, Kildare, Ireland; 5 Department of Health Science, Clinical Psychology, University of Damascus, Damascus, Syria; 6 Institute of Psychology, University of Münster, Münster, Germany; PLOS: Public Library of Science, UNITED STATES OF AMERICA

Natural disasters, such as floods, storms, heat waves, and droughts, are becoming more frequent and intense due to human-induced climate change, leading to significant disruptions in communities worldwide [1]. While these events pose immediate threats to physical safety, their long-term psychological impact is equally profound: Prevalences of 15–38% have been reported for a range of mental ill-health conditions following natural disasters, including posttraumatic stress disorder [2], depression [3], generalized anxiety [3], and prolonged grief [4]. These estimates are consistent globally [5,6] and remain high even years after the events [7]. Natural disasters may furthermore exacerbate the vulnerability of already marginalized groups such as individuals living in poverty or with a disability [8]. The urgency to establish reliable estimates of the public health consequences is underscored by recent projections of additional 14.5 million deaths and $1.1 trillion extra costs in healthcare systems worldwide due to climate-induced impacts [9]. In the aftermath of major disasters, there is a surge in mental health research aimed at understanding and mitigating mental health consequences. However, despite growing awareness, disaster mental health research faces persistent methodological and structural challenges. Many existing studies represent promising pioneering efforts to estimate the overall mental health burden in affected populations. However, there is significant room for improvement in the scientific rigor to better understand the impacts of disasters and derive more practical implications.

## Enhancing mental health research in post-disaster settings

Some challenges of post-disaster research are not unique to the particular context. Lack of validated translations of scales, culturally appropriate cut-off scores for such scales, and limited open access practices are common. Even when translated and validated measures for specific mental disorders are available, their application can be complicated by varying levels of mental health literacy and research interests may conflict with the practical need to efficiently identify a wide range of potential mental health concerns among affected individuals. Other barriers, such as logistic difficulties with obtaining in-nation ethical approval for research, low response rates, insufficient resources for large-scale interview-based studies, and lack of flexible funding for rapid study implementations are likely to remain, even when best practices are established.

**Fig 1:**
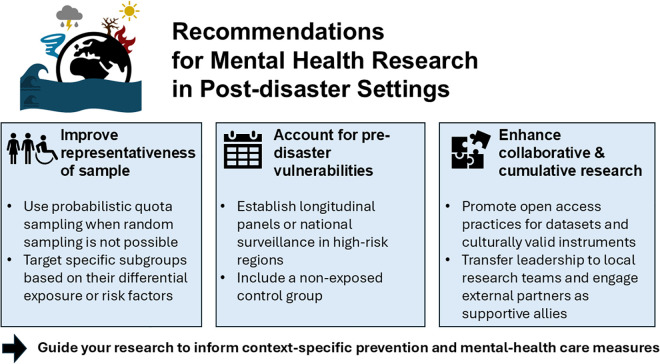
Recommendations to improve quality and interpretability of post-disaster mental health research.

However, there are also key factors we can influence to enhance research practices for improved, meaningful data quality (see Fig 1). First, samples in post-disaster research often lack representativeness of the overall affected population, as studies tend to focus on specific subgroups, convenient samples or the most severely affected areas. Random selection of participants is often impeded by restricted access to affected regions, particularly in remote areas, due to damaged infrastructure and ongoing relief efforts. Legitimate research interests can moreover conflict with more practical obstacles in disaster settings such as the creation of safe housing. Probabilistic quota sampling represents a cost-effective option in contexts where random sampling is not feasible to ensure that key characteristics of the population are adequately represented in the sample. The restriction to specific subgroups should be guided by their distinct experience or impact of the disaster, for example socioeconomically marginalized groups [8]. Research on such groups can help improve their mental health care to buffer the interaction between disasters and existing vulnerabilities.

Second, post-disaster assessments of affected individuals only allow for limited conclusions on the disaster’s impact, especially when pre-disaster prevalences of mental disorders in the affected regions are unknown. This is particularly concerning given that many natural disasters occur in post-conflict settings and that certain other traumatic events, such as gender-based violence, become more prevalent in the aftermath of natural disasters [10]. Many studies moreover do not account for participants’ pre-existing vulnerabilities that may have exacerbated the disasters’ effects, such as prior mental health conditions, or unprotected living environments. Given the unpredictability of natural hazards and hence difficulties in implementing pre-post studies, studies should include a non-exposed control group with comparable characteristics to account for additional influences on prevalence estimates. The inclusion of control samples represents a long-term investment necessary to enhance the interpretability and utility of post-disaster data. Given that the frequency and severity of disasters are projected to increase significantly over the next years [1], it is crucial to establish a rigorous database now. By doing so, we can optimize disaster response measures in the future and ultimately reduce long-term costs and human suffering. A key advancement would moreover be the implementation of longitudinal panel studies or national surveillance projects in regions at high risk for (both sudden- and slow-onset) natural hazards to understand mental health trajectories and examine the interplay of individual and social factors. Some panel studies have already been implemented [11,12], but future projects require enhanced mental health data and increased assessment periods.

Third, cumulative and collaborative research should be enhanced, for example by adopting open access practices, sharing datasets, and providing translated assessment tools. Most research funding is concentrated in high-income countries, which often enables external teams to conduct studies. From an estimated global investment in mental health research of $18.5 billion in 2015–2019, 88.5% of grants were based in high-income countries [13]. Yet, it is crucial to recognize that individuals from affected communities provide invaluable insights. Local research teams should be supported and included as they have the knowledge to tailor research to the cultural context and the specific needs of their communities and may have established human resources in the disaster area as well as trusted relationships with local communities and authorities.

## From insight to action: Enhancing prevention strategies in disaster contexts

Insights from high-quality disaster research on context-specific predictors and influencing factors of mental health complaints can guide the design and adaptation of evidence-based prevention measures which currently lack efficacy [14]. It is furthermore noteworthy that, although natural disasters disproportionately impact low- and middle-income countries [15], their representation in intervention studies remains limited [14]. It is therefore crucial to translate insights from disaster studies into effective interventions that are tailored to the specific needs of these countries. Overall, research on effective mental health prevention strategies should include both pre-disaster and post-disaster measures. Pre-disaster measures may involve community resilience-building programs, mental health literacy campaigns, and training of first responders to recognize and respond to early signs of psychological distress. Post-disaster measures can include rapid access to psychosocial support services, targeted psychological first aid, and long-term mental health care for affected individuals. Mitigating the mental health consequences of natural disasters by strengthening social cohesion and existing community networks may be particularly helpful in disadvantaged communities.
